# Pro-Environmental Sustainability and Political Affiliation: An Examination of USA College Sport Sustainability Efforts

**DOI:** 10.3390/ijerph18115840

**Published:** 2021-05-29

**Authors:** Jonathan M. Casper, Brian P. McCullough, Danielle M. Kushner Smith

**Affiliations:** 1 Parks, Recreation, and Tourism Management, North Carolina State University, Raleigh, NC 27695, USA; 2 Health & Kinesiology Department, Texas A&M University, College Station, TX 77843, USA; brian.mccullough@tamu.edu; 3 Exercise and Sport Science, University of North Carolina at Chapel Hill, Chapel Hill, NC 27599, USA; daniellesmith@unc.edu

**Keywords:** politics, fan behavior, collegiate sport, environment, sustainability, sport ecology

## Abstract

Political ideology is one of the most powerful predictors of perceptions about environmental sustainability and related behaviors. The purpose of this study was to investigate how sport fans’ sustainability-specific values, perceptions, and norms related to awareness, engagement, and influence of USA collegiate sport sustainability efforts based on political affiliation, accounting for age and gender. Data were collected using an online survey distributed to season ticket holders after the 2019 college football season that featured three sponsored sustainability initiatives at each home game. Multivariate analysis of variance and chi-square difference tests found that self-identified Democrats reported significantly higher pro-environmental values and norms, but sustainability program engagement, sponsored initiatives awareness, and influence of initiatives on behavior were politically neutral. Path analysis found that ascription of responsibility was a significant predictor of sustainability-related engagement and behaviors for both Independents and Republicans. The results and discussion sections highlight how academics and practitioners can account for political affiliation when creating campaign messaging for environmental initiatives.

The perceived importance of environmental sustainability varies among Americans. In most cases, opinions and actions regarding climate change, environmental protection, and sustainable behaviors have been influenced by politics and an individual’s party identification [1,2] Findings from presidential elections indicate that attitudes toward the environment and climate change significantly impacted the electorates’ chosen candidate [3,4]. The politicization of the natural environment and climate change is at an all-time high. For instance, conservatives are more skeptical of climate change and less willing to combat the causes of climate change than their progressive counterparts [1]. As a result, political ideology and affiliation are some of the most powerful predictors of an individual’s perceptions of climate change and related sustainable behaviors [5]. Thus, static environmental worldviews and political ideologies are significant challenges to overcome or neutralize to influence adults’ pro-environmental behaviors [6]. 

Those who seek to promote and change environmental behaviors have often felt frustrated with polarization, feeling that there is no way to educate conservatives and influence such behaviors (i.e., Republicans; [1]). Initial empirical support indicates that sport teams and organizations may provide a neutral platform in which fan sport team affiliation can be leveraged to promote pro-environmental concern among populations whose political affiliations bias their views (e.g., to be climate change skeptics and disengaged from sustainable behavior calls to action) [7,8].Specifically, the United Nations Sport for Climate Action Framework [9] was intended to engage the sport sector in climate action and be used as a social platform to engage and encourage broader and captive audiences. As an initial step in the framework, sport organizations should be authentic messengers, participate in legitimate environmental sustainability programs, and then engage their sport fans to follow suit. This approach is consistent with calls by academics [10,11]. and follows a logical progression of the environmental sustainability movement within the sport sector [12]. To date, there are over 215 signatories of the Sports for Climate Action Framework, most of whom are advancing their environmental initiatives by engaging their fans [11].

Signatories and other sport organizations actively develop fan engagement strategies and marketing campaigns to increase fans’ sustainable behaviors [13,14]. These campaigns are designed to increase fans’ awareness of environmental issues and encourage both at-event and post-event sustainable behaviors. Previous research has provided a deeper understanding of fans’ receptivity toward sustainability [7,8,15,16]. McCullough [8] found that political affiliation and beliefs toward environmental sustainability could be neutralized by leveraging fan identification. However, more recently—perhaps associated with the increased politicization of climate change and the environment—conservative sport fans may feel alienated and may reject calls to be environmentally responsible [7]. 

With these mixed results and the lack of direct examination, it is necessary to examine the influence that political affiliation has on environmental sustainability fan engagement campaigns. Specifically, a more concerted study can determine whether sport is an appropriate platform to engage target populations in conversations and behavioral changes to combat climate change as claimed by the United Nations. This understanding is also essential to inform academic approaches and the practical applications to properly design such campaigns for maximal impact. For example, this line of inquiry adds to the application of social identity theory in an area of sport management research that examines converging identities (sport, politics, environmental) and their influence in sport contexts to motivate specific behaviors. Thus, the purpose of this study was to investigate how college football fans’ political affiliation related to environmental values and beliefs, awareness of athletic-sponsored sustainability programs, and in-game initiatives, as well as at-home and at-game sustainability behaviors. 

## 1. Literature Review

Sport is an optimal platform to raise awareness around environmental sustainability [17]. However, there is a need to determine whether sport affiliation and the context of sport can alter a target market’s view around environmentally sustainable behaviors and influence potential future behaviors. Yet, variables such as political affiliation, gender, and age are still a new topic within sport ecology literature [18]. Thus, this exploratory study investigates the influence of political affiliation on environmental sustainability within a sport context. 

Sport and the natural environment engage in an ongoing bidirectional relationship that McCullough and colleagues [18] outlined when conceptualizing sport ecology. They note the vagueness and ambiguity of sustainability that have prevailed in various industries. As a result, businesses, including sport organizations, can co-opt the term and engage in greenwashing [19]. However, the sport sector has greatly advanced its environmental efforts from starting with signaling efforts to convey environmental values to fans through recycling programs, on-site renewable energy sources, and community engagement efforts (e.g., beach clean ups, e-waste recycling events). The sport sector has gotten more advanced by diffusing best practices within individual organizations and across the sport sector by sharing best practices [12]. 

Researchers in sport management have also followed the environmental trends within the sport sector [20]. Specifically, researchers have paid more attention on the environmental impact of sport organizations and events [21]. Specific to this study, sport has an impact on the natural environment through the production and consumption of the sporting experience whether that is by spectators or participants. In particular, [22] explored the environmental impact of a collegiate athletic department. They found that travel (spectators, athletes) and energy consumption were the highest contributing factors to the department’s overall carbon footprint. Yet, the political nature of environmental sustainability has begun to creep into sport [7] and more focus is necessary to help sport practitioners understand how to maximize their environmental efforts in light of this emerging issue. In the space below, we explore how political affinity related to environmental values and beliefs, awareness of athletic-sponsored sustainability programs, and in-game initiatives, as well as at-home and at-game sustainability behaviors. 

### 1.1. Environmental Sustainability and Politics

#### 1.1.1. Political Ideologies

Past psychological and behavioral research has identified static environmental worldviews and political ideologies as significant challenges to influencing adults’ pro-environmental behaviors. Worldviews and political ideologies typically solidify when individuals are in their early 20s [23] and can be powerful drivers of environmental perceptions and behaviors in various contexts [24,25]. As environmental sustainability, especially climate change, has become increasingly politicized, polarization has fallen along political lines, with conservatives being more skeptical of climate change and less willing to act against it than liberals [1]. Political ideology is the most powerful predictor of environmental sustainability perceptions and related behaviors [5].Many researchers have pointed to this finding to explain why environmental sustainability communications often fall short among conservatives [26] or encourage more skepticism and inaction [27,28]. 

#### 1.1.2. Gender, Age, and Politics

Researchers have found that both age and gender are critical socio-economic factors when examining environmental contexts [29,30]. Vicente-Molina, Fernández-Sainz, and Izagirre-Olaizola [31] found that gender plays an important role in shaping determinants of pro-environmental behavior. Gendered differences have been observed concerning both sustainability and political influences. From a sustainability perspective, scholars have examined different gender attributes; however, little attention has been given to theories and empirical evidence to explain pro-environmental behaviors by taking gender differences into consideration [32,33]. Female environmental behaviors are presumed to stem from their care and concern for the intersection of future generations and the environment [34,35]. In their annual review of environment and resources, Meinzen-Dick et al. [36] expand on this assumption using multiple dimensions of the impact of gender on sport, including closeness to nature, focus on sustainability, right to resources, means and opportunities to exploit resources and adoption of sustainable practices. Scholars argue that women are closer to nature biologically, socially, materially, and ideologically and also note that adoption of resources may be gendered due to lack of knowledge, labor, or financial resources [36]. McCright and Dunlap [26] have extensively documented what they term “the white male effect,” in which white males are more likely to be skeptical of environmental sustainability and resistant to mitigation measures. Additionally, some studies suggest that women exhibit some types of pro-environmental behavior at higher rates than men [37,38,39,40], but findings are not robust over time and across geographic areas around the world [40,41]. When looking at age, the literature suggests that younger people are typically more concerned about the environment than older generations [30]. However, there is conflicting research around the attitudes and behaviors of younger generations, e.g., millennials, around environmental sustainability. A recent study looking at American age and generation differences regarding environmental concerns found that neither age nor generational cohort correlated with support for “severity of environmental losses nor support for future actions to prevent them” [42]. This particular study also included political orientation as a predictor of environmental attitudes and future actions. Their findings suggest that political and value orientations may be superior factors to analyze over generational cohorts and age [42].

### 1.2. Sport as a Platform

Message framing is one of the most common strategies for overcoming social barriers to environmental sustainability engagement and action. Framing the appeals for addressing climate change to fit conservatives’ ideology has proven successful in changing conservatives’ attitudes and behaviors. For instance, conservatives expressed more pro-environmental attitudes and behaviors when such behaviors were framed as an obligation to one’s nation [43]. Additionally, conservatives’ skepticism about climate change science decreased if the solution to climate change was described as supporting capitalism [44]. In other words, conservatives can become more pro-environmental when doing so aligns with morals and values that are consistent with their worldviews. Though these findings are encouraging, more avenues are needed to deliver pro-environmental messages in appealing ways to those most likely to ignore them (i.e., conservative, white males). 

American football is watched by all political orientations and genders (~65% male) [45]. Football is especially attractive and popular with male conservatives. “It is America’s corporate sport, rising in our industrial heartland. The basis of football is its machinery—11 individuals subjugating themselves to the greater good of the team” [45]. Further, college football is a sport that skews to high voting turnout Republican fans, and the game’s focus on tradition and toughness has made it a bastion for conservative values [46]. Within a sport context, McCullough [8] found that more politically conservative fans would be more likely to engage in environmental initiatives if the sport organization asked them to behave in such a manner. Thus, theoretical and empirical evidence exists, and it is worthwhile to explore further whether or not there are different responses among people with differing political affiliations.

### 1.3. Fan Affinity and Behavioral Asks

Sport researchers have regularly measured social identification as a core construct of sport consumer behavior [47] through the lens of sport team identification [48,49,50]. “(Sport) Team identification (Team ID) primarily refers to the extent that an individual maintains a psychological connection with a sporting team and the emotional value he or she attaches to team support” [47] Individuals with strong Team ID typically derive self-esteem from their team doing well [51], and Team ID has been shown to positively impact fan perceptions of cause-related marketing initiatives [52]. Sport identity is constructed in similar ways to the socialization of any other group identity (e.g., political identity, gender identity). These sport fandom attributes create a promising yet understudied avenue to overcoming ideological barriers to climate change engagement and inaction.

Casper et al. [53] found that a majority (68%) of college sport fans desire sustainability initiatives. Sport organizations that have implemented environmental-focused fan engagement campaigns have seen positive social (sustainable behavior change) and financial (support for corporate sponsors) returns on investment [15,16,53,54,55]. For example, sport organizations can influence the sustainability actions in which fans engage at sporting events (e.g., composting). Such sustainability campaigns also influence personal norms and behaviors in everyday life [54,56]. These researchers all conclude that points of attachment (e.g., fan identification) are significantly influential to sport fans receiving campaign messages and inspiring sustainable behaviors by socially norming. Therefore, an athletic group identity associated with fandom (e.g., acting environmentally responsibly because it is what X fans do) may operate in an almost identical way to political identity (e.g., I support climate change action because I am a Democrat). It is then necessary to examine the influence of political identity on sport fans’ receptivity (i.e., attitudes) and consequential behaviors.

### 1.4. Values, Beliefs, Norms, and Behaviors

Researchers have used various theoretical frameworks to understand sustainable behaviors. The value–belief–norm theory (VBN) [57] is one of the more common frameworks used within the literature. Stern and colleagues leverage aspects of universal theory of human values [58], norm activation [59], and the new environmental paradigm [60] to understand the concepts of values, beliefs, and norms as predictors of behaviors or behavioral intentions. Researchers have successfully used this framework, in whole or in part, to understand the antecedents of sustainable behaviors and behavioral change in sport [15,16,53,54,55,61]. For example, Trail and McCullough’s work integrated the VBN with other theoretical frameworks to create the sustainable sports consumer evaluation model (SSCEM) [55]. Casper and colleagues alternatively exclusively use the VBN framework to examine the influence of various factors on sustainable behaviors and behavioral intentions. In the sections below, we explore the concepts of values/beliefs impact on norms and subsequent norms influence sustainable behavioral outcomes. 

**Values.** Schwartz and Bilsky [58] defined values by five critical features of the concept from the literature. Those five values included “(a) concepts or beliefs, (b) about desirable end states or behaviors, (c) that transcend specific situations, (d) guide selection or evaluation of behavior and events, and (e) are ordered by relative importance” [58]. The influence of values on norms and subsequent behaviors have been tested across a variety of contexts, including ethical behavior and organizational citizenship behaviors [62], social norms [63], pro-environmental behaviors in everyday life [15,64] and at sporting events [53,54,55]. Specifically, environmental values are informed and formed by knowledge and understanding of environmental issues [65]. This study investigates those values through the lens of understanding (lack of) environmental sustainability, perceptions of climate change, and perception of the importance of sustainable behaviors.

Individual sustainable behaviors are positively influenced by understanding or knowledge of the pertinent issue [65]. For example, DeYoung [66] found that individuals engaged in more environmentally sustainable behaviors when they knew about the issue and consequences of inaction. Ramsey and Rickson [67] classify knowledge into two categories: declarative choice (i.e., ecological knowledge) and procedural knowledge (i.e., trade-off knowledge). Ecological knowledge reflects an individual’s familiarity with the extent and causes of environmental degradation or climate change. As a result, knowledge of environmental sustainability and understanding of climate change demonstrates an individuals’ comprehension of environmental problems. Trade-off knowledge examines the degree to which an individual expresses the importance of engaging in ecological or sustainable behaviors and the consequences of inaction. Understanding or knowledge of environmental sustainability, climate change, and the importance of sustainable behaviors informs an individual’s values toward the natural environment and consequently informs social and societal norms.

**Norms.** While values can be individualistic, as discussed in the section above, values can also be collective. Collective values, or norms, can influence the motivational domain [58]. That is to say, at the basic level, individuals form their values based on their experiences. Then, collectively, individual values accumulate and form social norms and expectations of the behavior of specific entities. For example, ascription of responsibility [68] was conceptualized to frame how individual values inform the expectations that a “specific entity resolves an issue that is within their duty upon which to act” [56]. Dunlap and Van Liere [60] and Stern [69] have used ascription of responsibility within the new environmental paradigm and the VBN framework. Stern uses ascription of responsibility as part of the norming phase of the VBN framework. Ascription of responsibility has been assessed in sport ecology research within the collegiate sport and national sport fan contexts [15,53,54,56]. 

Similar to ascription of responsibility, perceptions of control result from the social power domain [58]. Perceptions of control are the direct consequence of collective values that influence and inform reward resources. Within collective norms, desired behaviors and fulfilling the social norm of a group are behavioral rewards for maintaining the larger group’s collective identity Thus, with higher levels of social norms, barriers to behavior change are more readily overcome to achieve specific environmental behaviors [61,70]. 

**Behaviors.** Individuals engage in consumer behaviors differently depending on the context and medium [71]. The success of sustainability campaigns is dependent on the organization and its identified key performance indicators (KPIs) [55]. Trail and McCullough posit that the goals of campaigns can be basic levels of awareness or recall that a campaign exists to engage in desired behaviors (i.e., sustainable behaviors) in a variety of contexts. The varying KPIs and subsequent campaign results will vary because fan segments will be at various stages, from generating general awareness to education on behavioral engagement or advocacy for desired behaviors, per Trail and McCullough [55]. For instance, the one expected campaign outcome may include that consumers engage with a campaign beyond the context of an event to learn more on social media. The resulting consumer engagement on social media is different from personal behavioral change [72]. Thus, a campaign can have various outcomes in its campaign messaging’s call to action. By way of example, a campaign can ask for the audience to seek more information by visiting a specific website or following the entity or campaign on social media. Similarly, the campaign can ask the audience to recycle while at the event, take sustainable transportation to the venue, or engage in these behaviors in their everyday lives (e.g., at work, at home, at play). The organization evaluates the KPIs based on the campaign messaging and can evaluate success based on the KPIs of interaction with the campaign on social media and self-reported behaviors. 

To expand upon the prior literature on the influence of political identification and environmental sustainability efforts in sport [7,8] and the influence of sustainability campaigns in sport [15,53,54,55,73], we seek to examine the influences of fans’ political affiliation on sport-specific sustainability campaigns to assess how political affiliation, as well as interactions with age and gender, may relate to sport-specific sustainability, this study was informed by prior literature and the VBN [69]. As a result, we are guided by the following research questions:

RQ1: How does political party affiliation relate to environmental sustainability values and beliefs (lack of understanding of environmental sustainability, perceptions of climate change, and the importance of sustainable behaviors)?

RQ2: How does political party affiliation relate to environmental sustainability behavioral norms (ascription of responsibility, perception of behavioral control)?

RQ3: How does political party affiliation relate to at-home and at-event sustainable behaviors, engagement with the athletic-led sustainability program, recognition of sponsored sustainability initiatives, and influence on initiative-specific behavior change?

RQ4: When comparing political party affiliation, what values best predict norms and what norms predict at-home and at-event sustainable behaviors, engagement with the sustainable program, and recognition of sponsored sustainability initiatives?

## 2. Method

### 2.1. Participants and Procedure

The study protocol was approved through the human subjects review board at the lead author’s institution. Data were collected using an online survey hosted by Qualtrics. Email invitations were sent out by the client (Athletics Department) to football season ticket holders one week after the final home game of the 2019 season. Participants who completed the survey were offered an incentive to enter a draw for a $100 gift certificate. The survey was open for one week with a follow-up reminder sent by the athletic department five days after the initial email; a total of 548 surveys were completed. 

### 2.2. Instrumentation

The survey included demographics (age, gender, income, and affiliation with the university—alumni or non-alumni) and two political items (political party affiliation and political ideology: “please select what best describes your political ideology”; 7-point scale, 1 = liberal, 4 = moderate, 7 = conservative). Respondents were also asked about their football game attendance for the 2019 season.

Items and constructs related to lack of understanding sustainability, perceptions of climate change, the importance of sustainable behavior, ascription of responsibility, and perceptions of control are listed in Table 1. Three items used to assess understanding of sustainability originated from Pritchard, Funk, and Alexandris [74] but were adapted to align with environmental sustainability initiatives similar to Trail and McCullough [55,61]. Participants’ perceptions of climate change (i.e., beliefs concerning climate change) were assessed using five items adapted from Dunlap [75]. The importance of sustainable behavior was assessed using three items adapted from Trail and McCullough [55] specific to this study’s context. Ascription of responsibility was assessed using three items adapted from Casper et al. [53] and focused on the campus, athletic department, and individual ascription of responsibility. Perceptions of control were assessed using three items from Schwartz and Bilsky [58]. 

At-home self-reported sustainability behavior was measured using four items. We specifically asked about behaviors related to the athletic department’s in-game environmental initiatives related to at-home recycling, water usage, and sustainable transportation. Specifically, respondents were asked, “Approximately what percentage of the time do you engage in the following activities AT HOME? [Recycling, Composting, Water Conservation, Use environmentally-friendly transportation (e.g., rideshare, environmentally-friendly transportation)]. Similarly, at-event self-reported sustainability behavior was measured using three items related to recycling, composting, and sustainable transportation to the game.

We used four items to assess sustainability program engagement. The first item asked whether they were aware of the program (1 = yes or 0 = no). The second item asked whether respondents could identify the program’s purpose with one correct answer (“university name” athletics sustainability program) and three dummy items. This item was later re-coded to 1 = correct or 0 = incorrect. The third item asked about engagement in the program based on six digital and in-game activities (later recoded to 1 = engaged with at least one or 0 = not engaged). The final items asked about their awareness of the athletics program as a signatory on the United Nation’s Sports for Climate Action Framework (1 = yes or 0 = no). The construct for analysis was created by totaling the number from each of the four items (range = 0–4).

There were three sustainability-focused sponsored initiatives featured over the football season. The first was related to aluminum cup recycling with activations in the tailgating zones as well as in stadium. The second initiative focused on water conservation and was primarily activated in stadium and included a text message pledge. The third, focused on sustainable transportation, was conducted via social media and offered a free mobile app. For each of the three initiatives, the survey asked whether respondents were aware of the initiatives (1 = yes or 0 = no). The construct for analysis, termed initiative awareness, was created by totaling the number of initiatives respondents were aware of (possible range = 0–3).

If a respondent indicated that they were aware of an initiative, they were presented with two additional items to assess behavioral awareness and influence. For awareness items were “The XXXX initiative increased my awareness of what can be [recycled, water conservation, or alternative modes of transportation].” For influence, the items were “The XXX initiative influenced me to increase the percentage of [waste I recycle in my daily life, conserve more water in my daily life, or increase my use of alternative modes of transportation].” Each item was measured on a 7-point Likert-type scale from strongly disagree to strongly agree. 

As an additional measure of awareness, respondents were asked to identify the corporate sponsor for each of the three initiatives. Each item had the correct sponsor and three “dummy” sponsors, which were later recoded to 1, indicating correct sponsor identification, or 0, indicated incorrect sponsor identification.

## 3. Data Analysis

Data were analyzed with IBM SPSS Statistics 25 and AMOS 26. The first step was a descriptive analysis and an examination of the data for normality (i.e., skewness and kurtosis), applying critical values of less than +/−2.0 for skewness and less than +/−3.0 for kurtosis [76]. Confirmatory factor analysis (CFA) was conducted to assess constructs based on multi-item scales (lack of understanding sustainability, perceptions of climate change, importance of sustainable behavior, ascription of responsibility, and perceptions of control). CFA assessed the reliability and validity of constructs based on factor structure, composite reliability (CR), and average variance extracted (AVE) [77]. 

Analysis for RQ1–3 included a multiple analysis of variance (MANOVA) for significant differences in construct means. Analysis of variance (ANOVA) was used to investigate the mean differences between the three initiative awareness and three influence items. Since environmental sustainability behaviors have been associated with age and gender differences [29,30], all analyses conducted accounted for interactions between political parties, age, and gender. A chi-square analysis was conducted to examine if political party affiliation, age, and gender related to correct sponsor identification of the three initiatives. 

For RQ4, a multigroup path analysis using political party as the grouping variable examined how values (based on knowledge, perceptions, and importance) predict norms (ascription of responsibility, perceptions of control) which predict behaviors (at-home, at-event, program engagement, and initiative identification). The overall model was assessed using the following fit indices: Comparative Fit Index (CFI), Tucker–Lewis Fit Index (TLI), and Root Mean Square Error of Approximation (RMSEA). According to Hu and Bentler [77], fit index values of CFI and TLI above 0.90 and RMSEA values less than 0.05 are considered acceptable. 

## 4. Results

The average age of the respondents was 53.33 years old (standard deviation (*SD)* = 14.28). For analysis purposes, age was categorized into younger adults (18–39 years, *n* = 100), older adults (40–64 years, *n* = 278), and seniors (65 years and older, *n* = 132). The respondents were mostly male (*n* = 398, 76.7%). A majority (85.2%) of the respondents had a household income over $75,000 annually, and 62.4% were alumni. A total of 84.4% of the respondents attended at least five of the possible seven home football games in the 2019 season. The sample populations in this study closely matched athletic department internal records of football fan demographics (i.e., age, gender, alumni status, income). 

Since this study sought to compare respondents based on politics, we first established how political party affiliation (party affiliation) related to political ideology (“Please select what best describes your political ideology”; 7-point scale, 1= liberal, 4 = moderate, 7 = conservative). An ANOVA test using Tukey’s post hoc test revealed significant (*F* = 267.70, *df* = 2, *p* < 0.001) differences between all three party affiliations. Democrats were more liberal (Mean (*M)* = 2.42, *n* = 155), Independents were more moderate (*M* = 3.97, *n* = 174), and Republicans more conservative (*M* = 5.72, *n* = 134). The results support the use of party affiliation as a categorical variable. 

RQ 1, 2, and 3 related to how political party affiliation, age, and gender relate to the constructs measuring values, behavioral norms, at-home/at-sport event behaviors, and engagement and identification of sustainability programs and initiatives. Results specific to political affiliation are shown in Table 2. When analyzing the constructs based on political affiliation, there were significant differences between each party for perceptions of climate change, the importance of sustainable behaviors, and ascription of responsibility. There were significant differences between Democrats and Republicans for understanding sustainability, at-home behaviors, at-event behavior, and engagement with the sustainability program. For gender, significant differences included females reporting higher ascription of responsibility (*F* = 4.48, *p* = 0.035), more awareness of the sustainability program (*F* = 4.689, *p* = 0.031), and higher at-home (*F* = 4.42, *p* = 0.036) and at-event sustainable behaviors (*F* = 7.61, *p* = 0.006). Older adults reported significantly higher perception of control (*F* = 3.61, *p* = 0.028); otherwise, there were no other age-related differences. No significant interaction effects were present. 

Table 3 shows the ANOVA results examining the awareness and influence of each of the three sustainability initiatives specific to political parties. Overall, there were no significant differences between political parties except for the recycling initiatives having significantly more influence for Democrats compared to Independents. Females were more influenced by the recycling initiative (*F* = 10.14, *p* = 0.023), but no other differences were found based on gender. There were no significant age differences or interactions. 

A chi-square analysis examined whether respondents accurately identified the correct sponsor for each sustainable initiative based on political party, age, and gender (see Table 4). Results show that the percentages were fairly equal, and no significant differences were found based on political party and gender. The older adults were significantly (*p* < 0.05) more likely to correctly identify the three initiative sponsors than young adults and seniors.

For RQ4, a multigroup path analysis examined how values (based on knowledge, perceptions, and importance) predict norms (ascription of responsibility, perceptions of control) which predict behaviors (at-home, at-event, program engagement, and initiative recognition) based on political party affiliation. Results of this path analysis are shown in Table 5. Overall, the path model showed good fit (Democrat *n* = 155, Independent *n* = 134, Republican *n* = 174; chi-square = 56.609 [*df* = 36]; CFI = 0.982, TLI = 0.933, RMSEA = 0.035).

## 5. Discussion

The purpose of this study was to empirically investigate how college football fans’ political ideology related to environmental values and beliefs, awareness of athletic-sponsored sustainability programs, and in-game initiatives, as well as at-home and at-game sustainability behaviors. Previous researchers have explored the influence of dissenting values [55] and even political affiliation [7] on environmentally sustainable campaigns and behaviors within a sport context. These previous studies provide a strong foundational understanding of the possible influence of these consumer attributes (i.e., political affiliation, environmental values/beliefs) on the receptivity to sustainable campaign messages and the desired outcomes of such campaigns (e.g., behavioral change). Our findings from this study show that values related to understanding, perceptions, and importance of sustainability differ based on political lines, which Kellison and Cianfrone [7] also found in their qualitative study. Additionally, norms, assessed through ascription of responsibility, show that Democrats are significantly more pro-environmental than Independents and Republicans; while this is expected in broader society, it is also evident among identified college sports fans. A key finding is that while there are differences in pro-environmental values and norms, behaviors and awareness of sponsored initiatives and their associated influence on sustainability behavior are less differentiated by political party affiliation. The sections below focus on some of the major findings and implications based on the research questions.

Specifically, RQ1 sought to understand how political party affiliation relates to environmental sustainability values and beliefs. There were significant differences between Republicans and Democrats specific to their lack of understanding of sustainability, where Republicans demonstrated a significantly higher level of lack of understanding sustainability. There were significant differences among all political affiliations concerning perceptions of climate change and the importance of sustainable behaviors. In both cases, Democrats had significantly higher perceptions of climate change and a greater understanding of the importance of sustainable behaviors than Independents and Republicans. Similarly, Independents had a significantly higher perception of climate change and a greater understanding of the importance of sustainable behaviors than Republicans. Republicans significantly differed and had the lowest perceptions of climate change and understanding of the importance of sustainable behaviors compared to Independents and Democrats. These findings are consistent with prior research that found that Republicans had a more difficult time explaining climate change than their Democrat counterparts and were less motivated to make behavioral modifications [78].Similarly, RQ2 explored how political party affiliation related to environmental sustainability behavioral norms—particularly, ascription of responsibility and perception of behavioral control. Our results found significant differences among all three political affiliations specific to the ascription of responsibility. Democrats held significantly higher levels of ascription of responsibility compared to Independents scores were significantly higher than Republicans. This research is consistent with prior literature that suggests that Democrats are more likely to see collective action, or collective responsibility, as necessary to address climate change [79]. However, there were no significant differences among or between the political affiliations based on perceptions of control. These results suggest that participants across all political affiliations believed that they had control over their ability to protect the environment, act in environmentally friendly ways, and act sustainably. This finding diverges from previous research that found that political affiliation was a significant predictor of individual levels of climate change perceptions and control to act sustainably [80]. One potential explanation for this may be the context in which participants were asked these questions and the possible association with a sport property with a deep commitment to environmental sustainability. 

In RQ3, we examined whether political party affiliation related to at-home and at-event sustainable behaviors, engagement with the athletic-led sustainability program, recognition of sponsored sustainability initiatives, and influence on initiative-specific behavior change. Our data found significant differences in self-reported behaviors (at-home and at-event) and sustainability program engagement between Republicans and Democrats. That is, Democrats reported a significantly higher likelihood to engage in at-game and at-home sustainable behaviors than Republicans. However, Democrats reported that they engaged in at-home sustainable behaviors 54.69% of the time and at-event sustainable behaviors 41.21% of the time. Participants from all three political affiliations did not differ based on their awareness of the three sponsored sustainability initiatives. That is, all participants had similar awareness levels of the various sponsored initiatives. This finding is encouraging, given the politicization of climate change and environmental sustainability, and that the integration of a sponsor on a particular program did not deter a fan based on their political affiliation [81,82]. At best, these findings suggest that all fans are equally aware of the various initiatives, indicating that lack of awareness is not associated with political affiliation but may just be a barrier in the type of sponsor communication. Moreover, the recycling sponsor was correctly identified most frequently, followed by the water conservation initiative sponsor, and last, the sustainable transportation sponsor. 

Further, for those aware of the initiatives, political affiliation was not related to awareness of the importance of specific sustainability behaviors or the initiative’s influence on their behavior. Our findings demonstrate that all three sponsored initiatives were effective, with behavioral awareness and influence means high across party lines. This finding is encouraging because the campaign delivered on developing awareness and seeking to influence sustainable behaviors for all fans despite the possibility for political divisiveness. Additionally, no political differences for sponsor identification were found in our results, showing that the brand did not necessarily impact potential behavior and influence. Finally, age was the only differentiator among sponsorship identification, demonstrating that adults had a significantly higher percentage of correct identification than seniors. 

Building on these findings, RQ4 compared political party affiliation to what values best predict norms and subsequently what norms predict at-home and at-event sustainable behaviors, engagement with the sustainable program, and recognition of sponsored sustainability initiatives. Our results suggest that respondents’ perceptions about the importance of sustainability were the best predictor of ascription of responsibility across all party lines. In contrast, the perception of climate change was a significant predictor for Independents and Republicans only. This result suggests that while environmental initiative awareness was equal across all three political affiliations, political affiliation did inform the perceptions of climate change and the resulting ascription of responsibility. This finding is consistent with other research examining environmental attitudes and environmental action in consumer behavior research [83] and sport literature [84]. These studies suggest that political affiliation predicts environmental attitudes and responses to address climate change. 

Further down the model, RQ4 found that ascription of responsibility was a stronger predictor of behaviors and awareness than perceptions of control. Although ascription of responsibility was a significant predictor of at-home, at-event, and program awareness for Independents and Republicans, it only significantly predicted at-home behaviors for Democrats. This finding is consistent with prior studies [53] in which ascription of responsibility was demonstrated to be a significant predictor of at-home and at-game behavioral intentions. However, Casper and colleagues did not explore the segmentation of these behaviors based on demographic characteristics or political affiliation. Our findings here further support the necessity to examine or evaluate the influence of political affiliation on ascription of responsibility and the influence that affiliation has on at-home and at-game behaviors.

Further, Kellison and Cianfrone [7] found that politically conservative fans felt alienated when sport organizations promoted environmentally sustainable messages and initiatives. Their finding is an important aspect to consider as more fans may see sport as becoming increasingly more politicized [85] and a significant barrier for sport practitioners to implement environmental sustainability initiatives into their organization [86] in fear of a negative fan response. Thus, environmental messages and campaigns ought to be tested beforehand to avoid such perceptions and to assess the level of responsibility, and even expectations of stakeholders, to address certain aspects of sustainability. For example, sport practitioners can use focus groups or survey fans to evaluate the effectiveness of sustainability messaging and whether it will resonate to encourage the desired outcome or whether fans will have an adverse reaction. This will allow researchers and practitioners to track the success of such campaigns and changes in environmental attitudes, values, and behavioral outcomes.

We also found that females reported significantly higher ascription of responsibility and behaviors (e.g., at-home, at-event) than males. This is consistent with prior literature around gender themes and the environment, specifically around females’ focus on sustainability and the means and opportunities to adopt sustainable practices [36]. Since initial gendered literature was surrounding only environmental behaviors and not in a sport context, this is significant in understanding the implications for gendered sustainability programs for sport organizations.

## 6. Conclusions

### 6.1. Managerial Implications

As sport sustainability efforts and initiatives seek to inform and influence fans regarding pro-environmental behaviors, there may be a concern that these efforts will be either ignored or rejected by those less pro-environmentally inclined, and in many cases, correlated with political affiliation [5]. The findings from this study may help quell such concerns at least related to the athletics-sponsored sustainability activities. Such initiatives may be an effective communication and education activity highlighting sustainability and climate change issues in a non-political fashion. Specifically, in their constant search for new revenue sources, athletics departments may focus on brands supporting an environmental message. Additionally, athletics events or games also serve as a setting for all fans to practice pro-environmental behaviors, and in many cases, they are rewarded for them through sponsored activations. Our results provide some initial evidence that sport events may serve as an ideal educational medium (or platform) to influence behavior in broader society. These activations may also serve as a benefit, and possible entertainment, to non-traditional fans such as females and older adults, who seem to respond positively to such messaging. 

### 6.2. Limitations and Future Directions

Despite this study’s novel approach to explore the influences of political ideology related to environmental values and beliefs, awareness of athletic-sponsored sustainability programs, and in-game initiatives, and at-home and at-event sustainability behaviors, there are limitations to our available design. Expressly, we were limited by the client with whom we worked. As such, we were limited in our data collection and used a cross-sectional study design. In these instances, the primary limitation is that the exposure and outcomes are assessed simultaneously [87]. In this case, we assessed the environmental beliefs and values alongside an awareness of environmental initiatives and the resulting influences of these initiatives on sustainable behaviors. Future researchers should if able, utilize a longitudinal study design with a pre-exposure and a post-event questionnaire. This would allow researchers to assess the actual influence of the sporting organization, event, or sponsor. Additionally, we were not privy to a mechanism to determine individual participants’ actual behaviors in this study. As a result, we relied on self-reported behaviors for both at-home and at-game behaviors. 

It should be noted that the concept of sustainability is particularly vague and misunderstood [16,21]). Specifically, consumers may have varying views of the term, what it means to them, and how to respond to those issues, whether through calls to action or self-motivated actions. That being said, researchers should examine the ambiguity of such concepts and how consumers interpret them, and how other differing identities may influence these perceptions (e.g., political affiliation, fan identification, religious identification). 

Moreover, future research should further examine the influence that political affinities and values have on the emerging social issues of our time that are replicated within the context of sport. Specifically, researchers should consider the influence of—or control for—political affinity in assessing environmental sustainability issues in sport. This may also extend to other social problems deemed political (e.g., police reform, Black Lives Matter). Longitudinal studies should also be explored to assess the participants’ values and environmental beliefs similar to the design utilized by Trail and McCullough [16]. 

### 6.3. Contributions

This study contributes to further insight into the role of political ideology, receptivity, and action related to sustainability using sport. While the political polarization of values, norms, and most behaviors was evident in the sample, awareness and influence of specific sustainability initiatives proved to be more equally received regardless of political party. Therefore, the messages are still being received by those who may be less pro-environmental, so sport may be a viable platform for environmental education and behavior change. The role of ascription of responsibility provides some meaningful avenues for education and influence for those less pro-environmental. The more a fan learns about the need, the reason, and their responsibility, the more likely they will act on such behaviors.

## Figures and Tables

**Table 1:** Items and Constructs.

Item/Construct	*M*	SD	Std. Factor Loading	AVE
**Lack of Understanding of Sustainability**				0.69
I don’t understand what the term “sustainable” means when referring to the environment	2.31	1.51	0.75	
I don’t understand what the term waste diversion means	2.97	1.71	0.63	
I don’t understand what climate change means	1.83	1.39	0.55	
**Perceptions of Climate Change**				0.93
Climate change is real	5.76	1.71	0.71	
Humans are responsible for climate change	5.21	1.89	0.84	
Climate change is impacting my community	5.15	1.77	0.81	
Climate change is the most serious global issue facing the human race	4.52	2.11	0.81	
Climate change can be addressed without hurting the economy	4.71	1.89	0.55	
**Importance of Sustainable Behaviors**				0.90
It is very important to act sustainably	5.88	1.26	0.78	
It is very important to protect the environment	6.28	1.05	0.58	
It is very important to reduce our impact on climate change	5.79	1.57	0.81	
**Ascription of Responsibility**				0.95
CU Boulder has a responsibility to integrate environmentally sustainable practices into their operations	5.75	1.38	0.78	
CU Athletics has a responsibility to integrate environmentally sustainable practices into their operations	5.7	1.44	0.79	
Every individual has a responsibility to integrate environmentally sustainable practices into their behaviors	5.78	1.44	0.76	
**Perceptions of Control**				0.89
I have no control over protecting the environment	2.17	1.36	0.68	
I have no control over acting in environmentally friendly ways	1.75	1.01	0.85	
I have no control over acting sustainably	1.82	1.07	0.84	

**Table 2:** MANOVA—Differences in Constructs Based on Political Party Affiliation.

Construct	*F*	*p*-Value	*η*2	Democrats Mean (*n* = 153)	Independents Mean (*n* = 170)	Republicans Mean (*n* = 131)	Sig. Group Differences *
Lack of Understanding Sustainability	2.72	0.05	0.14	2.19	2.40	2.58	b
Perceptions of Climate Change	40.40	0.00	0.18	6.16	5.00	4.00	a, b, c
Importance of Sustainable Behavior	26.03	0.00	0.11	6.57	6.03	5.38	a, b, c
Ascription of Responsibility	20.23	0.00	0.08	6.39	5.76	5.13	a, b, c
Perceptions of Control	1.14	0.32	0.01	5.25	4.81	4.96	none
At-Home Sustainable Behavior	9.71	0.00	0.04	54.69	45.46	40.67	b
At-Game Sustainable Behavior	4.84	0.01	0.02	51.21	44.81	42.40	b
Sustainability Program Engagement	5.42	0.01	0.03	2.77	2.68	2.48	b
Sustainability Initiative Awareness	0.90	0.41	0.00	1.18	1.14	1.17	none

* Note. Indicates significant (*p* < 0.05) differences between groups based on Tukey’s post hoc test. a = Democrats and Independents, b = Democrats and Republicans, and c = Independents and Republicans.

**Table 3:** ANOVA—Awareness and Influence of Sustainability Initiatives Based on Political Party.

Construct	*F*	*p*-Value	*η*2	Democrats Mean (*n*)	Independents Mean (*n*)	Republicans Mean (*n*)	Sig. Group Differences *
Recycling Awareness	2.16	0.12	0.01	5.01 (*n* = 152)	4.71 (*n* = 167)	4.67 (*n* = 130)	none
Recycling Influence	1.26	0.28	0.01	4.29 (*n* = 152)	3.92 (*n* = 168)	3.99 (*n* = 130)	none
Water Conservation Awareness	4.22	0.02	0.09	5.28 (*n* = 25)	4.21(*n* = 38)	4.42 (*n* = 38)	a
Water Conservation Influence	1.70	0.19	0.04	4.92 (*n* =25)	4.18 (*n* = 38)	4.29 (*n* = 38)	none
Sustainable Transportation Awareness	0.64	0.53	0.01	4.48 (*n* = 61)	3.83 (*n* = 54)	4.15 (*n* = 27)	none
Sustainable Transportation Influence	0.69	0.50	0.01	3.66 (*n* = 61)	3.39 (*n* = 54)	3.89 (*n* = 27)	none

* Note. Indicates significant differences between groups based on Tukey’s post hoc test. a = Democrats and Independents.

**Table 4:** Chi-Square Table Examining Identification of Sponsors for Each Sustainability Initiative.

Item	Democrats	Independents	Republicans	χ2 (*p*)	Young Adults	Older Adults	Seniors	χ2 (*p*)	Females	Males	χ2 (*p*)
Recycling Sponsor–Correct Identification	90.3%	90.8%	85.8%	2.256 (0.324)	20.6%	56.0%	23.4%	14.62 (0.000)	88.2%	89.4%	0.139 (0.709)
Water Conservation Sponsor–Correct Identification	26.5%	41.2%	32.4%	5.871 (0.053)	13.5%	45.9%	14.6%	9.90 (0.007)	13.4%	14.8%	0.140 (0.708)
Transportation Sponsor–Correct Identification	16.8%	10.4%	10.9%	3.421 (0.181)	29.7%	54.7%	12.5%	6.77(0.034)	13.4%	12.6%	0.064 (0.800)

**Table 5:** Multigroup Path Analysis Examining Values, Norms, and Behaviors Based on Political Party.

		Standardized Regression Value		
Predictor Variable	Dependent Variable	Democrats	Independents	Republicans
Lack of Understanding Sustainability	Ascription of Responsibility	−0.04	−0.02	−0.02
Perceptions of Climate Change		0.07	0.34 *	0.30 *
Importance of Sustainability		0.76 *	0.48 *	0.60 *
Lack of Understanding Sustainability	Perceptions of Control	0.03	−0.11	0.09
Perceptions of Climate Change		0.06	0.04	0.17
Importance of Sustainability		0.11	0.163	0.18
Ascription of Responsibility	At-Home Sustainable Behavior	0.23 *	0.35 *	0.39 *
	At-Event Sustainable Behavior	0.12	0.34 *	0.37 *
	Sustainability Program Engagement	0.14	0.28 *	0.30*
	Sustainability Initiative Awareness	0.14	0.10	0.12
Perceptions of Control	At-Home Sustainable Behavior	0.07	−0.08	−0.02
	At-Event Sustainable Behavior	0.13	−0.05	−0.03
	Sustainability Program Engagement	−0.08	−0.13	0.10
	Sustainability Initiative Awareness	−0.08	−0.01	−0.05

Note. * indicates a significant (*p* < 0.05) path.

## Data Availability

Data is available by contacting the corresponding author.
